# What roles does physical activity play following the death of a parent as a young person? A qualitative investigation

**DOI:** 10.1186/s12889-022-14542-6

**Published:** 2023-01-31

**Authors:** Jane Williams, Neil Howlett, Gillian W. Shorter, Julia K. Zakrzewski-Fruer, Angel Marie Chater

**Affiliations:** 1 Centre for Health, Wellbeing and Behaviour Change, Institute for Sport and Physical Activity Research (ISPAR), University of Bedfordshire, Polhill Avenue, Bedford, MK41 9EA UK; 2 School of Psychology, University of Bedfordshire, Luton, LU1 3JU UK; 3 Department of Psychology, Sport and Geography, University of Hertfordshire, Hatfield, AL10 9AB UK; 4 Centre for Improving Health Related Quality of Life, Queens University Belfast, School of Psychology, Belfast, BT9 5BN UK; 5 Drug and Alcohol Research Network, Queens University Belfast, School of Psychology, Belfast, BT9 5BN UK; 6 Centre for Behaviour Change, University College London, 1-19 Torrington Place, London, WC1E 7HB UK

**Keywords:** Physical Activity, Exercise, Parental Bereavement, Death, Grief, Social Support, Resilience

## Abstract

**Background:**

Physical activity benefits physical and mental health. However, limited research investigates if physical activity can improve outcomes from the grieving process following the death of a parent.

**Methods:**

Semi-structured interviews were conducted with 14 individuals (*n* = 8 female; age *M* = 31.2 years), who had experienced the death of a parent when they were aged between 10 and 24 years old, using retrospective recall. Data were analysed inductively using thematic analysis.

**Results:**

Six *themes* were identified. Physical activity was seen as; 1) ‘*Therapeutic’*; providing an 2) ‘*Emotional Outlet’* and created a strong sense of 3) ‘*Social Support’*. Alongside it 4) ‘*Builds Confidence’*, and led to 5) ‘*Finding Yourself’* and 6) ‘*Improved Health and wellbeing’* (physical and psychological).

**Conclusion:**

Physical activity has the potential to provide positive experiences following a parental bereavement. It can provide a sense of freedom and was seen to alleviate grief outcomes, build resilience, enable social support and create a stronger sense of self. Bereavement support services for young people who have experienced death of a parent should consider physical activity as a viable intervention to support the grieving process.

## Introduction

It is estimated that in the UK there are over 41,000 children and young people who experience the death of a parent each year [1]. Grief manifests itself in a variety of outcomes, such as depression, anxiety, and aggression [2–8]. As physical activity can have a positive impact on mental health [2, 3, 5], it is important to understand whether it could benefit grief outcomes. The current guidelines for physical activity are a minimum of 150 minutes of moderate (i.e. walking) or 75 minutes of vigorous (i.e. running) intensity aerobic activity a week, paired with muscle strengthening (i.e. weights) activity 2 days per week [9, 10].

Various types of physical activities have shown positive benefits for a variety of mental health outcomes within young people in the general population. Previous research has shown that physical activity can support children and adolescents with depression [5, 11, 12] and anxiety [3, 13, 14]. Adolescents (*N* = 11,110) with greater physical activity levels have been found to have lower depressive symptoms [3]. Activities such as Mixed Martial Arts (MMA) and combat sports can reduce aggression, providing a controlled outlet for children and young people [6]. MMA can also increase self-control skills, and reduce hostile thoughts [7]. In addition to children and adolescents physical activity has been shown to reduce symptoms in adults with clinical and non-clinical anxiety or depression [2, 15]. Participating in leisure time physical activities is linked to lower levels of anxiety within adults [16]. Whilst participating in physical activity with others helped to reduce isolation felt by widows, aged 35–64 [17, 18]. It further provides opportunities to meet new people, helping to reduce isolation and increase a sense of social support [19]. Participating in physical activity can help develop resilience to stress and overcome adversities [2]. In particular, a review suggests that outdoor interventions can help build resilience in at-risk children [20]. This body of research emphasises the range of benefits that physical activity can have on mental health outcomes and the possible benefit it may have on grief outcomes.

A systematic review investigating the roles of physical activity in supporting individuals who have been bereaved, provides evidence to suggest that physical activity may be an effective support mechanism [21]. Looking specifically at young people who have experienced the death of a parent there is evidence to suggest that physical activity can provide benefit. Individuals felt that physical activity offered an escape from emotions [22, 23], an opportunity to create friendships [22], providing social support to decrease loneliness [24, 25], reduced post-traumatic stress disorder [26] and built family cohesion [23]. However, the lack of homogeneity between these studies, in terms of methodology and outcomes measured limits the conclusions. There is limited focus primarily on the role of physical activity, with studies reporting the benefit of physical activity as a secondary finding [22, 24, 25, 26]. Further research investigating the role of physical activity for individuals who have experienced parental bereavement is needed as the review highlighted just one qualitative study, with a primary focus on physical activity benefits for young people who have experienced bereavement [23]. Furthermore, just one intervention which aimed to support childhood traumatic grief in young people who had experienced the death of a parent, used outdoor physical activity (i.e. canoeing) in combination with counselling [24]. Therefore, there are no interventions with a primary focus on investigating the role of physical activity on parental bereavement. A recent study on the experiences of parental bereavement, suggests that physical activity may be a suitable alternative to talking therapies [8].

The current study aimed to understand what roles physical activity may have had in a young person’s life following the death of their parent. The objective was to gain insight into personal experience of participating in physical activity whilst grieving and overall perceptions of using physical activity to benefit grief outcomes. Interviews were done retrospectively, to minimise psychological distress and account for an adjustment period following the death of a parent. This insight can help to inform future bereavement support services that may consider including physical activity as an intervention option.

## Method

### Design

A phenomenological qualitative approach using semi-structured interviews allowed for the generation of in-depth data. As two members of the research team (AMC, GWS) have experienced the death of a parent, two researchers (JW, NH) acted as bracketers to alleviate any preconceptions which may create bias in the data in those who have either experienced or not experienced the death of a parent [27]. The consolidated criteria for reporting qualitative research guidance (COREQ-32) [28], was used to report this study.

### Participants

Eligible participants were recruited using multiple strategies (social media, snowballing). The inclusion criteria was adults (over 18 years) who experienced the death of a parent as a young person (10–24 years; using the WHO definition [29]), 5 years or more prior to the interviews. Participants were interviewed 5 years or more to account for time to adjust to the death of a parent. A total of 14 (six males, eight females) individuals participated, aged from 21 to 41 years old (*M* = 31.2, *SD* = 6.6). The death of a parent was experienced between 11 and 24 years old (*M* = 17.9, *SD* = 4.1), the mean time since death was 13.1 years (*SD* = 3.7). No participant experienced the death of both parents between the ages of 10–24, however, some revealed that both parents had died by the time of interview. The death of a dad *(n* = 7) and mum (*n* = 7) were equally experienced, and these terms have been used in this paper to reflect the parental terms used in the participant’s narrative. Participant details are found in Table 1.

**Table 1:** Participant and bereavement information for those interviewed about the roles physical activity plays in the lives of young people following the death of a parent

Pseudonyms	Age in years (at interview)	Parent who died	Age at bereavement	Cause of death	^a^Expected/ unexpected death	Religion	Ethnicity	^b^Physically Active	Days active (30 minutes or more)
Marie	39	Dad	20	Illness	Unexpected	Catholic	British	Fairly	4 Days
Ben	33	Dad	18	Natural	Unexpected	Protestant	British	Active	6 Days
Rebekah	25	Mum	16	Illness	Unexpected	No religion	British	Fairly	3 Days
Laura	21	Mum	11	Illness	Unexpected	No religion	British	Fairly	2 Days
Jack	25	Dad	14	Illness	Expected	No religion	British	Active	7 Days
Kate	25	Mum	18	Illness	Expected	No religion	British	Fairly	4 Days
Jim	28	Mum	17	Illness	Expected	Christian	British	Active	7 Days
Greg	Asked not to share	Dad	Asked not to share	Illness	Unexpected	Asked not to share	Other	Fairly	3 Days
Zara	41	Mum	23	Illness	Expected	Christian	British	Active	5 Days
Adam	30	Mum	13	Illness	Expected	No religion	British	Active	7 Days
Chris	28	Dad	21	Illness	Unexpected	No religion	British	Inactive	0 Days
Claire	40	Mum	22	Illness	Unexpected	Christian	British	Active	7 Days
Louise	28	Dad	13	Illness	Unexpected	No religion	Other	Fairly	3 Days
Gail	38	Dad	24	Illness	Unexpected	No religion	British	Inactive	0 Days

^a^ Expected/ Unexpected death: Unexpected death is either sudden or earlier than anticipated; expected death has been anticipated due to illness or disease [30].

^b^Physically active: Physical activity that met the Sport England criteria for active (150 + minutes MVPA per week), fairly active (30–149 minutes MVPA per week) or inactive (< 30 minutes MVPA per week) at time of interview (MVPA = Moderate to vigorous physical activity)

### Materials

Participants received an information sheet detailing the nature of the study and an informed consent form. To reduce emotional distress at the onset, a brief questionnaire collected details relating to parental death using tick boxes rather than asking the participant to write which parent died and how. These tick boxes included demographic information (age, religion, ethnicity), details of bereavement (which parent had died, cause of death, if it was expected), and physical activity levels (currently physically active, member of a sports team and number of days in the last week they were active for 30 minutes or more). A semi-structured interview schedule was used to guide the interviews (see Table 2). Questions considered participants own individual experiences of participating in physical activity and perceptions of how physical activity could support others who are grieving. The interviewer recorded a field notebook, however, due to different modes of interview (in-person, by phone) this was not included in analysis. Two dictaphones (Tescam Dr-05) set to record at 44.1 kHz were used to audio record interviews.

**Table 2:** Questions used in semi-structured interview to determine the roles physical activity might play in the lives of young people following the death of a parent

Eliciting questions	
1. How did sport or physical activity fit in with your life after [x] died? ***Prompt****: Were you a member of…Sports teams/ after school/ outside school clubs/ bike riding with friends/ running/ gym?* ***Prompt****: How about before their death?*	
2. How did being involved in physical activity make you feel back then? ***Prompt:****[Release stress/ blow off steam/ escape from other pressures?]* ***Prompt:****What was PE like for you?*	
3. What are your thoughts on physical activity or sport helping individuals through the grieving process? ***Prompt:****What could be the benefits [*i.e. *sense of release]*	
4. What types of sport or physical activity do you think would be the most beneficial? ***Prompt****: Team sport/ competitive/ physical contact/ outdoors/ belonging?*	

### Procedure

Participants contacted the researcher via email to express interest in the study, and if eligible, an interview date was arranged. Interviews were held between March to September 2018. Following reading the information sheet and providing written consent, participants completed the brief questionnaire and gave both written and verbal consent for the interviews to be recorded. Interviews were held at; University of Bedfordshire campus (*n* = 3); phone call (*n* = 3); other university campus (*n* = 3); participant’s home (*n* = 2); local café (*n* = 2); participant’s workplace (*n* = 1) and lasted between 24 and 63 minutes (*M* = 44.04; *SD* = 12.82). Where possible to reduce participants distress, the interviews took place in a bright open space, with access to fresh air and refreshments available. Following a safeguarding protocol, both participants and the researcher were able to pause or stop the interview if they felt distressed, with the researcher mindful of emotional and physical distress due to the sensitive nature of the topic. Based on guidance from mental health and suicide experts, participants received a detailed de-brief, including signposting to bereavement support services and a follow up call the following day. Due to the sensitive nature of this topic, it was important for the interviewer to build a rapport with the participant, this was achieved by following active listening, non-verbal communication (i.e. eye contact), and being respectful of experiences [31], The interviewer (JW) was a white British female psychology postgraduate student, enrolled on a PhD programme, who had previous experience with qualitative research and interviewing. For researcher safeguarding, the interviewer participated in regular supervision meetings after each interview, with a HCPC registered health psychologist (AMC), who had extensive experience in qualitative research and had experienced parental bereavement at the age under investigation. Two other members of the team (NH and GWS) were also experienced in qualitative research methods. The interviewer (JW) and second coder (AMC) engaged in continuous reflexivity, with regular conversations and reflections of their role within the research and interpretation of the findings. This was supported by wider team meetings, to discuss the data and themes that were identified. Findings were also discussed with a small advisory group of people who had experienced the death of a parent (not included in the study) to strengthen credibility. Detailed process notes were taken throughout the study to transparently document the procedure, enhancing the study’s dependability and blind double-coding assisted with the study’s confirmability.

### Ethics

Ethical approval was given by the University of Bedfordshire ethics committee in December 2017 (Reference number: 2017ISPAR008). The British Psychological Society’s code of Ethics and Conduct was followed [32]. Any personal information was anonymised, and pseudonyms were given. Participation was voluntary and all participants had the right to withdraw from the study at any time. Confidentiality was maintained and participants were informed this would only be broken if there was considered to be a risk of harm to themselves or others. No incidences occurred where confidentiality was broken. To reduce the risk of psychological distress, participants were interviewed 5 years after the death of a parent, to gain a retrospective experience.

### Analysis

**Fig. 1:**
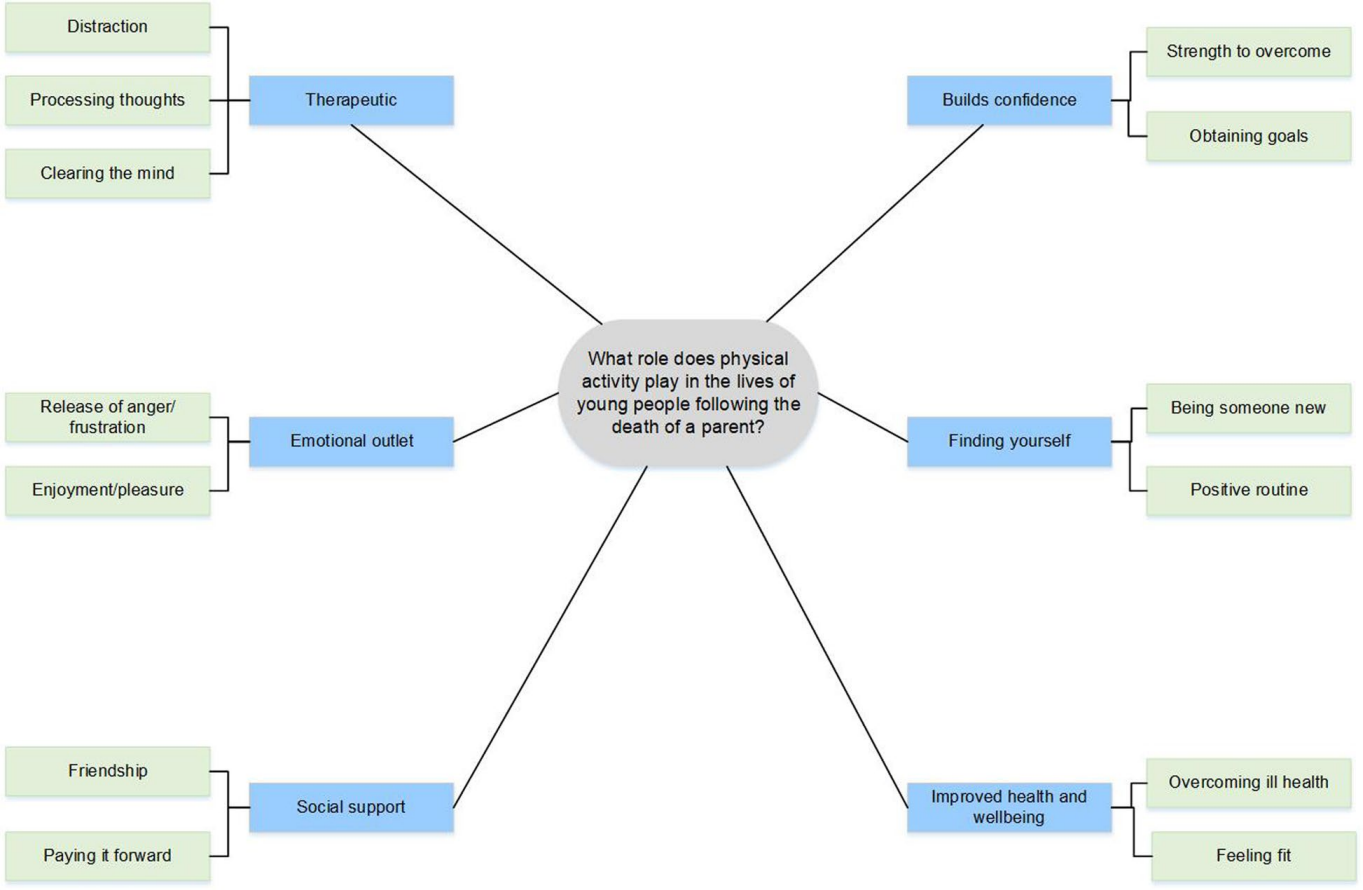
Central themes and sub themes of the roles of physical activity following parental bereavement

Interviews were transcribed verbatim (by JW), checked for accuracy (AMC) and coded (by JW & AMC) using Nvivo11 software [33]. Firstly, nodes were created using an iterative and inductive approach [34], followed by thematic analysis [35, 36] to create key themes presented in the thematic map in Fig. 1. As meaning is generated from the interpretation of the interviews not themes emerging from data, it is acknowledged based on current thinking that data saturation is subjective. However, no new ideas were interpreted from the later interviews (following interview 11) into the themes identified, and the acceptable sample size of ten plus three was reached [37]. The flexible approach of thematic analysis allowed the similarities across the dataset to be highlighted [38]. An iterative process was followed, undertaking four amendments to the coding framework before final themes were agreed by all coders (JW, AMC, GWS, NH). Any disagreement was resolved in discussion between coders.

## Results

Six themes were identified when answering the research question: ‘What roles does physical activity play in the lives of young people following the death of a parent?’: *1) ‘Therapeutic’; 2) ‘Emotional outlet’; 3) ‘Social Support’; 4) ‘Builds Confidence’; 5) ‘Finding yourself’ and 6) ‘Improved health and wellbeing’.* Themes and subthemes are presented in *italics*. Following a quote, the participant’s pseudonyms, age at bereavement and parent who died are presented in parenthesis.

### Physical activity characteristics for context

Engagement in physical activity following the death of a parent differed, as some participants engaged in the physical activity immediately after the loss. Continuing their routines, whereas others reported taking a break from their chosen activity, or waiting and finding physical activity some time after the bereavement. Participants reported, a variety of physical activities used to support their grief outcomes: badminton, dancing, gym, mixed martial arts, netball, running, rugby, outdoor activities (i.e. hiking), walking for leisure and walking for transport. The heterogeneity in the types and time taken to participate in physical activity following a bereavement, highlight how this process, like grief itself, is individualised, with no set process.

#### Theme 1 – ‘Therapeutic’

The data indicated physical activity was seen to have therapeutic qualities. It often provided a distraction from grief outcomes, allowing individuals time to process thoughts, and gave them a chance to clear their minds.

##### Distraction

Participating in physical activity provided a distraction from bereavement. It allowed time to escape from their grief and other life stresses which were experienced after the death of a parent. This was viewed as a welcomed and pleasant experience.

*“It [MMA] was nice, bit of an escape. Soon as I was there, soon as I was training, I sort of forgot about everything else. It was just my way of getting away from it all [ …] I think I actually got stuck in more during that period. For the next sort of few years and that kind of helped a lot. Kept me out of my head a little bit”* (Jim, 17 when mum died)

*“I love going to the gym. I feel like it is such a distraction. So, at the time I probably didn’t realise it was a bit of a distraction. Like put yourself towards something”* (Laura, 11 when mum died*)*

*“Just going to a match [badminton], you know, you’re totally focused on a game and it’s just something that takes you away”* (Kate, 18 when mum died)

##### Processing thoughts

Participants valued the time physical activity gave them to process their thoughts. There was a sense of freedom, where physical activity gave participants the time to be able to think. This processing of thoughts related both to being able to allow a stream of consciousness, but also to focus on thoughts related to the parent that had died.

*“Your thought process, you can just go from one train [of thought] to another to another. It’s so free and I think I do think about her a lot because there is no other space to do it in life. It’s busy”* (Zara, 23 when mum died)

*“It’s just being out on your own, you know. In the nicer parts of the country and sometimes not nice and putting on some music and just going off into your thoughts.”* (Greg, undisclosed age when dad died)

*“I think it’s just a bit of peace of mind, just being able to sort your thoughts out.”* (Kate, 18 when mum died)

##### Clearing the mind

Providing a distraction and processing thoughts allowed individuals a chance to clear their mind from any negative, dwelling, or stressful thoughts. This appeared to reduce rumination of the trauma of parental loss and provided a sense of ‘switching off’ from grief and attention is focused elsewhere.

*“You step on the training place or you step on the pitch and you just can’t for a second switch off, you can’t drift off, you can’t be worried about you know who’s watching from the side-lines. It [rugby] was just everything. It was yeah 100% focus on what you are doing”* (Adam, 13 when mum died)

*“There’s something about doing physical activity that makes being in your own head a lot nicer, more… kind of meditative”* (Greg, undisclosed age when dad died)

*“I mean it is sort of a good head clearing exercise and I do think there was something quite cathartic about it, particularly as I would just set myself loftier and higher goals all the time”* (Chris, 21 when dad died)

#### Theme 2- ‘Emotional outlet’

Physical activity allowed participants a way to express their emotions and provided an outlet for negative and positive emotional responses. It was felt to be a safe space where individuals were able to constructively channel their emotions without repercussions.

##### Release of anger/ frustration

Participants expressed how they became frustrated or angry following their parent’s death. The interviews emphasised how using physical activity enabled them to constructively channel their aggression and frustration in a positive manner.

*“There are loads of feelings you know, you can be angry in bereavement, and you can be really down and sad but actually if you get out and do physical activity both those things and all those things are going to feel better and going to be better* “*(*Zara, 23 when mum died)

*“Just being able to get that, you know, that worry and rumination and emotion out […] even if I have done something like drama or music, nothing’s compared as much as sport” (*Kate, 18 when mum died)

*“I was never a scrapper or fighter and stuff off the pitch [rugby] and I guess it gave me that release, that physical side of it was really good for me”* (Adam, 13 when mum died)

*“Positively sort of channelling all this weird energy that I had”* (Jim, 17 when mum died)

##### Enjoyment/ pleasure

Physical activity not only alleviated negative emotions; it also enabled the experience of positive emotions. By participating in physical activity, participants were able to enhance their mood, feel good about themselves, and reduce feelings of sadness. This wasn’t necessarily intentional, or something they were looking to achieve, but would often be linked to when they were feeling down.

*“It [running] would just lift my mood really. I wouldn’t feel as down about what happened to my dad or my life at the time”* (Ben, 18 when dad died)

*“I would say it [walking] felt good, it felt good but it’s not something I would have willingly done [exercise gained through work], so it’s good I was forced to”* (Greg, undisclosed age when dad died)

*“Naturally, you would start to look for something. I guess you don’t know, I don’t think I was doing it consciously, well I’m just going to do this and this. So, I don’t feel miserable”* (Louise, 13 when dad died)

#### Theme 3 – ‘Social support’

Participants noted the importance of social support after experiencing the death of a parent. Participating in physical activity, allowed those interviewed to develop friendships and the opportunity to pay it forward to benefit others through activity linked to charity work.

##### Friendship

The interviews highlighted how participating in physical activity reduced feelings of isolation and distance that can be felt following a bereavement. Participants created deeper bonds with friends and/or met new friends. Peers were able to provide support towards their grief, and helped to reduce loneliness, by participating in physical activity together.

*“They [bereaved individual] are just going to get into their own bubble, and they don’t want to talk to anyone and if they don’t have annoying friends who are like no let’s go breakdance, let’s go to my class or something then what does this child do then.”* (Louise, 13 when dad died)

*“I just started running for the club, went to training sessions and started to make friends there and realised, you know, this was really for me, this was something I felt like this was the sport that I really enjoyed”* (Ben,18 when dad died)

*“I did a get back into netball thing with my girlfriends and I really enjoyed that. We were all really busy workwise and it was a way of us kind of meeting up as well as doing an activity […] Honestly, it gave me something to do with my friends. You know I’d go to the gym with my neighbour, and I would go to netball with my girlfriends, and it was just something to look forward to”* (Marie, 20 when dad died)

##### Paying it forward

Of those interviewed, illness was the most common reason for the death of their parent. Participating in physical activity was seen as a good way to raise awareness and money to help support different charities which cater to those illnesses or for those who have experienced a similar loss. By paying it forward to charities, individuals had a greater sense of social purpose, and valued what they were giving to others.

*“It [charity runs] probably makes everyone feel better that everyone is running, and everyone is doing stuff for the exact same cause and to raise money and to try and not make that happen to someone in the future.”* (Rebekah, 16 when mum died)

*“I was craving the fitness and the pursuit of goals and the challenge of forcing myself to hit milestones or whether it was the kind of charity part of it that really motivated me but yeah fitness became a very important thing for me around that time.”* (Chris, 21 when mum died)

Raising money for charity and paying it forward was not at the forefront of all participants minds. One participant was motivated to run a marathon after the death of her Mum, but instead of doing it to raise money for a charity, she was doing it to positively focus on herself.

*“The first marathon I ever ran was a year after my mum died, and I think the focus; I didn’t raise any money. I just felt like I couldn’t do that I just wanted to do it for me and in the focus of training and having something to like work towards felt right to do somehow.”* (Zara, 23 when mum died)

#### Theme 4 - ‘Builds confidence’

The interviews highlighted how physical activity allowed participants to build confidence in themselves and their own capabilities. Self-confidence was seen to decrease after the death of a parent, and by participating in physical activity it gave participants the confidence to achieve goals, and the strength to overcome adversity faced.

##### Strength to overcome

Participants showed personal growth and developed resilience from the death of their parent. This growth was seen in how they approached physical activity, which enabled them to build up resilience and develop the strength to keep pushing themselves physically (i.e. running that extra mile) and emotionally (i.e. overcoming adversities). They felt nothing could come close to being as tough as the death of their parent, and if they can overcome that, they can overcome anything.

*“But again, for me at the tough times of that climb I really pulled on that like the strength of that I can get through the fact my mum has died and I can do this. So, I think there is definitely a physical, mental connection there for me.” (*Zara, 23 when mum died)

*“I mean I know its daft when you’re training at the gym it sometimes hurts a little, but I realised I was able to push myself further than before just because I had this new sort of lease of motivation”* (Jim, 17 when mum died)

##### Obtaining goals

Achieving goals helped individuals to develop self-confidence. The participants expressed how having a physical activity goal or something to strive or work towards enabled them to regain control during uncertain times.

*“I think for me it was a sort of goal setting thing where in quite a big way really it was about forcing myself to meet certain goals and thereby sort of demonstrate that I was in control of myself, I guess to potentially [control] my sort of emotions and my thought process as well as my bodily goals”* (Chris, 21 when dad died)

*“Personal bests have always, what I’ve seen myself as, and I like to go to races where I am the slowest runner and hanging on the back. I know a ton of people from the outside judge that as he is a bad runner, but I don’t see myself as that. I would like to be in bigger races where I am holding on to dear life”* (Jack, 14 when dad died)

#### Theme 5 - ‘Finding yourself’

Physical activity gave participants the opportunity to find themselves during a chaotic time. Grief caused shifts in thoughts, feelings, and action and physical activity enabled participants to become someone new, away from their grief. Finding a positive routine was important for individuals to find themselves, allowing them to regain some control in their life.

##### Being someone new

Each individual experienced a unique grief journey, however, it was clear that their grief response shaped the person they became. For some, participating in physical activity helped them to feel like themselves again, regaining a sense of normality. One participant had experienced issues with drug use after the death of his dad, which left him in a dark place, however after participating in physical activity (i.e. walking) he felt like a new person.

*“But when I joined the badminton team, I had people that I could relate to on a sporting level. Then I just became more myself again”* (Kate, 18 when mum died)

*“Think I just got to the stage that I just needed to start getting back into a routine and start getting back into my normal kind of life.”* (Rebekah, 16 when mum died)

*“I think before therapy and recovery my head wasn’t a very good place to be. But when I was exercising or when I was doing, you know, doing this kind of work, it would make it more bearable almost.”* (Greg, undisclosed age when dad died)

##### Positive routine

Physical activity was used to create positive routines. It was something which participants turned to, providing them with structure during a seemingly uncontrollable time. Some participants emphasised how they have always been physically active, only stopping after the death of a parent.

*“Gave me a real good structure you know and it taught me I needed to be independent and organised to be able to make sure it happened, which has stood me in good stead later in life and stuff”* (Adam, 13 when mum died)

*“At the point I was training and competing in martial arts anyways. But I think to be fair that was something I turned to more than ever”* (Jim, 17 when mum died).

*“I was quite active sort of around the point the bereavement took place and it did take a couple of years to actually get back into a routine to be physically active”* (Ben, 18 when dad died)

#### Theme 6 - ‘Improved health and wellbeing’

Grief not only affected individuals emotionally, but they also experienced physical reactions to the death of a parent. Physical activity allowed the participants to alleviate physical and emotional symptoms of grief, overcoming ill health and enhancing their mood.

##### Overcoming ill health

Improving health in general was noted as a benefit of being physically active. This benefit was described alongside supporting mental health and outcomes such as weight loss.

*“Basically, I had a couple of health scares I had a few issues with my health I realised that I was sort of shutting myself away a bit of the time and I just started to improve my health”* (Ben, 18 when dad died)

*“I felt healthier I could breathe more”* (Gail, 24 when dad died)

*“I think it is a regular break from depression. Where you would do 30/40 days of eight hours a day walking in a row. You know I went from being incredibly skinny when I was younger up until the age of 23/24, I couldn’t put on weight no matter how hard I tried. I was tall and very kind of thin and then all of a sudden after doing a few years of doing these letter deliveries with this heavy bags I just absolutely grew out, massively much bigger than I am now in healthy and unhealthy ways cos I did put on a lot of weight during a short period of time.”* (Greg, undisclosed age when dad died)

##### Feeling fit

During the chaotic period trying to adjust to life after a parental death, participants found physical activity allowed them to feel better, with physical fitness mentioned on a number of occasions. They recognised the release of endorphins helped to boost their mood, feel happy, relaxed, and in control when participating in physical activity.

*“So, joining a gym to me was like going back to what I know, what I enjoy, all the sport and fitness and all that side of things. It is keeping me fit”* (Claire, 22 when mum died)

*“I think by keeping fit and getting out the house and doing stuff is good for you”* (Laura, 11 when mum died)

*“Yeah, I felt I was in control of my experience, I was improving my health; it just felt like I had some more control over my life and my exercise”* (Ben, 18 when dad died)

## Discussion

Six themes were identified and collectively highlight the roles physical activity can play following the death of a parent. Physical activity is seen as *1) ‘Therapeutic’.* It provided *an 2) ‘Emotional outlet’* and created a strong sense of *3) ‘Social support’*. It also *4) ‘Builds confidence’* and individuals reported that it helped to *5) ‘Finding yourself’.* Ultimately, physical activity was perceived to have helped to *6) ‘Improved health and wellbeing’*, physically and psychologically.

Physical activity was therapeutic to participants during their grief, as it provided them with a distraction, giving them time to think and process their thoughts. It gave some clarity during an uncertain time. Similar findings have been reported elsewhere, [23] showing physical activity to be an escape from grief outcomes following the death of a parent. The current study highlighted that physical activity provided a constructive outlet for emotions, both positive and negative. Individuals were able to process feelings of depression, anxiety, and anger during physical activity, while it also brought them pleasure and joy. Research has found that physical activity can reduce negative emotions and boost positive emotions for aggressive behaviour in children [39], and for adolescents and adults who have experienced trauma (i.e. bereavement) [40–43] which further supports the findings of this study. The current findings provide support for a variety of physical activities supporting bereavement in young people following the death of a parent. However, the analysis in this study did not consider differences between types of activities, therefore, there remains question, of which type of physical activity, (e.g. rugby, running, MMA, contact vs non-contact) might most effectively benefit grief outcomes, and if there are dose responses based on duration in activity and frequency of performance. Future research should aim to address these questions.

Isolation is a common grief outcome which can be aided with social support [5, 44]. Findings from this research support this, showing participating in physical activity with peers or team mates provided beneficial support to individuals following the death of their parent, and helped to build new friendships. Research shows that being around like-minded people, and others that have experienced similar circumstances provides valuable support [24, 45, 46]. Whilst participants in this research benefited from peer support from those who hadn’t experienced parental bereavement, previous research [24] found that meeting others who had experienced bereavement, helped individuals realise that they are not alone in grief. This raises an important question as to what extent it is the physical activity that supports grief outcomes, versus the social support that may come with the activities. This is an area worthy of future investigation.

Post-traumatic growth was reflected in the subthemes of strength to overcome and obtain goals. One of the changes referred to by participants in this research was the strength to overcome new challenges by using physical activity to push themselves further and train harder to obtain goals. Obtaining goals can in turn increase self-confidence [47], which can help to develop overall physical and mental health. This appears to be an important mechanism of action for this population. Previous research has found similar findings, suggesting individuals who have been parentally bereaved may experience post-traumatic growth by building resilience and relationships [8].

Grief can not only have a psychological impact on individuals but also a physical impact. Physically, grief can be a risk factor for illnesses (i.e. cancer or heart disease) [48] and may leave individuals feeling exhausted [49]. During grief, the immune system can become impaired [50, 51] as a result of a stress-response and cortisol modules supressing the immune system [52]. Participants highlighted how physical activity was used to overcome or support physical illnesses which may have been triggered by their grief. Being physically active can boost fitness, which can strengthen the immune system [53, 54], and overall help individuals to feel fit and overcome ill health.

This paper provides valuable insight into the roles of physical activity following the death of a parent, bereavement organisations, who provide support to young people following the death of a parent, should be able to use these findings to offer physical activity services for support. Despite the valuable insight of this study, it is not without limitation. Qualitative data can be interpreted differently by members of the research team dependent on lived experience. Two analysts have experienced the death of a parent, therefore, it was important to ensure the use of bracketers (JW, NH), so that the personal experiences of the team who had been bereaved (AMC and GWS) did not bias data interpretation. Other limitations were the time taken for recruitment and transcription. Additionally, participants were not given their transcripts to provide further comments, therefore follow up information may have been missed. Individuals self-selected for the interviews, and were therefore, open to sharing their experiences and opinions. Each participant provided a generous amount of information during their interview and were positive about their experiences of using physical activity. While field notes were taken, direct observations were not always able to be made due to the varying nature of interviews (in-person vs on the phone). As the interviews were held retrospectively, with mean time since death 13 years ago, participants may have forgotten important information or key emotions felt. A further limitation of this study is the lack of culture diversity within the sample. Grief and their associated rituals differ between cultures, something that is not represented in this study. As many of the participants were physically active, they may have been more open to the benefits of physical activity and may not offer a fully representative view. Future research should aim to include those who are both active and not active, to widen the generalisability of such findings. Additionally, further research within this field should consider the age difference at time of bereavement, and investigate differences in the approach to participating in physical activity between age groups.

## Conclusion

This study extends the scientific knowledge of the roles of physical activity following a parental bereavement at a young age. It highlights that physical activity has the potential to be therapeutic, making it a viable option for bereavement support. It can provide a sense of freedom following loss, and alleviate grief outcomes such as isolation, depression, aggression and loss of control. Physical activity can further build resilience, enable social support, create a stronger sense of self and enhance perceived physical health and wellbeing. These findings can help the future development of bereavement support services for young people who have experienced death of a parent, which should consider physical activity interventions as an alternative or adjunct to talking therapies.

## Data Availability

Data is available from the corresponding author on reasonable request which will not conflict with the anonymity and confidentiality.

## References

[1] Child Bereavement Network (2016). Key estimated statistics on childhood bereavement.

[2] Fox KR (1999). The influence of physical activity on mental well-being. Public Health Nutr.

[3] McMahon EM, Corcoran P, O’Regan G (2017). Physical activity in European adolescents and associations with anxiety, depression and well-being. Eur Child Adolesc Psychiatry.

[4] McDowell CP, MacDonncha C, Herring MP (2017). Brief report: Associations of physical activity with anxiety and depression symptoms and status among adolescents. J Adolesc.

[5] Eime RM, Young JA, Harvey JT (2013). A systematic review of the psychological and social benefits of participation in sport for children and adolescents: informing development of a conceptual model of health through sport. Int J Behav Nutr Phys Act.

[6] Harwood A, Lavidor M, Rassovsky Y (2017). Reducing aggression with martial arts: a meta-analysis of child and youth studies. Aggress Violent Behav.

[7] Shachar K, Ronen-Rosenbaum T, Rosenbaum M (2016). Reducing child aggression through sports intervention: The role of self-control skills and emotions. Child Youth Serv Rev.

[8] Chater AM, Howlett N, Shorter GW, Zakrzewski-Fruer JK, Williams J. Reflections on Experiencing Parental Bereavement as a Young Person: A Retrospective Qualitative Study. Int J Environ Res Public Health. 2022;19(4):2083. 10.3390/ijerph19042083 PMC8872611 35206275

[9] Davies S, Atherton F, McBride M (2019). UK Chief Medical Officers’ Physical Activity Guidelines.

[10] World Health Organisation. Physical activity, https://www.who.int/news-room/fact-sheets/detail/physical-activity (2020), (Accessed 3 Mar 2022).

[11] Bailey R (2006). Physical education and sport in schools: a review of benefits and outcomes. J Sch Health.

[12] Janssen I, LeBlanc AG (2010). Systematic review of the health benefits of physical activity and fitness in school-aged children and youth. Int J Behav Nutr Phys Act.

[13] Ahn S, Fedewa AL (2011). A meta-analysis of the relationship between children’s physical activity and mental health. J Pediatr Psychol.

[14] Biddle SJH, Asare M (2011). Physical activity and mental health in children and adolescents: a review of reviews. Br J Sports Med.

[15] Rebar AL, Stanton R, Geard D (2015). A meta-meta-analysis of the effect of physical activity on depression and anxiety in non-clinical adult populations. Health Psychol Rev.

[16] Kim J, Chun S, Heo J (2016). Contribution of leisure-time physical activity on psychological benefits among elderly immigrants. Appl Res Qual Life.

[17] Kang HY, Yoo YS (2007). Effects of a bereavement intervention program in middle-aged widows in Korea. Arch Psychiatr Nurs.

[18] Yoo YS, Kang HY (2006). Effects of a bereavement intervention program on depression and life satisfaction in middle aged widows in Korea. J Korean Acad Nurs.

[19] Mason OJ, Holt R (2012). Mental health and physical activity interventions: a review of the qualitative literature. J Ment Health.

[20] Ungar M, Dumond C, Mcdonald W (2005). Risk, resilience and outdoor programmes for at risk children. J Soc Work.

[21] Williams J, Shorter GW, Howlett N, Zakrzewski-Fruer J, Chater AM. Can physical activity support grief outcomes in individuals who have been bereaved? A systematic review. Sports Medicine-Open. 2021;7(1):1–7. 10.1186/s40798-021-00311-z PMC8028581 33830368

[22] Brewer J, Sparkes AC (2011). Young people living with parental bereavement: Insights from an ethnographic study of a UK childhood bereavement service. Soc Sci Med.

[23] Brewer J, Sparkes AC (2011). The meanings of outdoor physical activity for parentally bereaved young people in the United Kingdom: Insights from an ethnographic study. J Adventure Educ Outdoor Learn.

[24] McClatchey IS, Wimmer JS (2012). Healing components of a bereavement camp: children and adolescents give voice to their experiences. J Death Dying.

[25] Zhao J, Chi P, Li X (2014). Extracurricular interest as a resilience building block for children affected by parental HIV/AIDS. AIDS Care.

[26] McClatchey IS, Vonk ME, Palardy G (2009). Efficacy of a camp-based intervention for childhood traumatic grief. Res Soc Work Pract.

[27] Rolls L, Relf M (2006). Bracketing interviews: Addressing methodological challenges in qualitative interviewing in bereavement and palliative care. Mortality.

[28] Tong A, Sainsbury P, Craig J (2007). Consolidated criteria for reporting qualitative research (COREQ): a 32-item checklist for interviews and focus group. Int J Qual Health Care.

[29] World Health Organisation. WHO and United Nations Definition of Adolescent - Public Health, https://www.publichealth.com.ng/who-and-united-nations-definition-of-adolescent/#:~{}:text=The%20World%20Health%20Organization%20%28WHO%29%20and%20the%20United,the%20Convention%20on%20the%20Rights%20of%20the%20Child (2017), (Accessed 3 Mar 2022).

[30] Hui D (2015). Unexpected death in palliative care: What to expect when you are not expecting. Curr Opinion Support Palliative Care.

[31] Hull M (2007). Building a rapport with patients. The Foundation Years.

[32] British Psycholgical Society. Code of Ethics and Conduct (2018) | BPS, https://www.bps.org.uk/news-and-policy/bps-code-ethics-and-conduct (2018), (Accessed 6 Feb 2019).

[33] QSR International Pty Ltd. (2020) NVivo (released in March 2020). https://www.qsrinternational.com/nvivo-qualitative-data-analysis-software/home.

[34] Thomas. (2006). Method notes a general inductive approach for analyzing qualitative evaluation data. Am J Eval.

[35] Braun V, Clarke V (2006). Using thematic analysis in psychology. Qual Res Psychol Aging.

[36] Braun V, Clarke V (2019). Reflecting on reflexive thematic analysis. Qual Res Sport Exercise Health.

[37] Francis JJ, Johnston M, Robertson C (2010). What is an adequate sample size? Operationalising data saturation for theory-based interview studies. Psychol Health.

[38] Clarke V, Braun V. Thematic analysis: a practical guide. London: SAGE Publications LtD.; 2022.

[39] Turpomanova T, Filkova S, Mincheva-Bolgurova P (2015). Innovative Sports Activities To Overcome the Aggressive Behaviour in the School - Aged Children. Res Kinesiol.

[40] Manger TA, Motta RW (2005). The Impact of an Exercise Program on Posttraumatic Stress Disorder, Anxiety, and Depression. Int J Emerg Mental Health.

[41] Newman CL, Motta RW (2007). The effects of aerobic exercise on childhood PTSD, anxiety, and depression. Int J Emerg Mental Health.

[42] Motta R (2016). The Role of Exercise in Reducing PTSD and Negative Emotional States. Psychology of Health - Biopsychosocial Approach.

[43] Lambert L, D’Cruz A, Schlatter M (2016). Using physical activity to tackle depression: the neglected positive psychology intervention. Middle East J Positive Psychol.

[44] Lamont E, Harris J, McDonald G (2017). Qualitative investigation of the role of collaborative football and walking football groups in mental health recovery. Ment Health Phys Act.

[45] Mitchell AM, Wesner S, Garand L (2007). A support group intervention for children bereaved by parental suicide. J Child Adolescent Psychiatric Nurs.

[46] Hung NC, Rabin LA (2009). Comprehending childhood bereavement by parental suicide: a critical review of research on outcomes, grief processes: and interventions. Death Studies.

[47] Weinberg RS, Robert S, Gould D. (2011). Foundations of sport and exercise psychology.

[48] Crunk AE, Burke LA, Robinson EH (2017). Complicated grief: an evolving theoretical landscape. J Couns Dev.

[49] Shear MK, Simon N, Wall M (2011). Complicated gried and related beraement issues for DSM-5. Depress Anxiety.

[50] Chen GT, Prigerson H (2005). Health behaviors associated with better quality of life for older bereaved persons. J Palliat Med.

[51] Balk DE (2011). Adolescent development and bereavement: an introduction. Prev Res.

[52] Nanthakumar C (2018). The benefits of yoga in children. J Integr Med.

[53] Nieman DC, Wentz LM (2019). The compelling link between physical activity and the body’s defense system. J Sport Health Sci.

[54] Lombardi G, Ziemann E, Banfi G (2019). Physical activity and bone health: What is the role of immune system? A narrative review of the third way. Front Endocrinol.

